# 7-Bromo-4-(7-bromo-3,3-dimethyl-1-oxo-2,3,4,9-tetra­hydro-1*H*-xanthen-9-yl)-3,3-dimethyl-2,3-dihydroxanthen-1(4*H*)-one ethanol solvate

**DOI:** 10.1107/S1600536809035922

**Published:** 2009-09-12

**Authors:** Chaomei Lian, Pingping Lu, Yulin Zhu

**Affiliations:** a School of Chemistry and Environment, South China Normal University, Guangzhou 510006, People’s Republic of China

## Abstract

The title compound, C_30_H_26_Br_2_O_4_·C_2_H_5_OH, was synthesized from the reaction between 5-bromo­salicybenzaldehyde and 5,5-dimethyl-1,3-cyclo­hexa­nedione. The crystal packing is stabilized by inter­molecular O—H⋯O hydrogen bonds and C—H⋯O inter­actions.

## Related literature

For the properties and applications of xanthenes, see: Gusak *et al.* (2000 ▶); Sato *et al.* (2008 ▶); Wang *et al.* (2005 ▶).
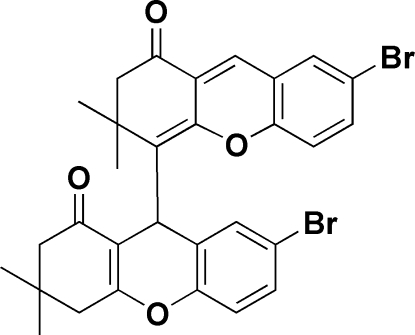


## Experimental

### 

#### Crystal data

C_30_H_26_Br_2_O_4_·C_2_H_6_O
*M* *_r_* = 656.40
Orthorhombic, 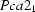

*a* = 15.016 (2) Å
*b* = 10.2459 (14) Å
*c* = 18.740 (3) Å
*V* = 2883.2 (7) Å^3^
*Z* = 4
Mo *K*α radiation
μ = 2.85 mm^−1^
*T* = 298 K
0.26 × 0.18 × 0.12 mm

#### Data collection

Bruker APEXII area-detector diffractometer
Absorption correction: multi-scan (*SADABS*; Bruker, 2004 ▶) *T*_min_ = 0.546, *T*_max_ = 0.710
14442 measured reflections
5155 independent reflections
3685 reflections with *I* > 2σ(*I*)
*R*_int_ = 0.041

#### Refinement

*R*[*F* ^2^ > 2σ(*F* ^2^)] = 0.039
*wR*(*F* ^2^) = 0.078
*S* = 1.01
5155 reflections
357 parameters
1 restraint
H-atom parameters constrained
Δρ_max_ = 0.33 e Å^−3^
Δρ_min_ = −0.27 e Å^−3^
Absolute structure: Flack (1983 ▶), 2379 Friedel pairs
Flack parameter: 0.020 (8)

### 

Data collection: *APEX2* (Bruker, 2004 ▶); cell refinement: *SAINT* (Bruker, 2004 ▶); data reduction: *SAINT*; program(s) used to solve structure: *SHELXS97* (Sheldrick, 2008 ▶); program(s) used to refine structure: *SHELXL97* (Sheldrick, 2008 ▶); molecular graphics: *SHELXTL* (Sheldrick, 2008 ▶); software used to prepare material for publication: *SHELXTL*.

## Supplementary Material

Crystal structure: contains datablocks global, I. DOI: 10.1107/S1600536809035922/jh2097sup1.cif

Structure factors: contains datablocks I. DOI: 10.1107/S1600536809035922/jh2097Isup2.hkl

Additional supplementary materials:  crystallographic information; 3D view; checkCIF report

## Figures and Tables

**Table 1:** Hydrogen-bond geometry (Å, °)

*D*—H⋯*A*	*D*—H	H⋯*A*	*D*⋯*A*	*D*—H⋯*A*
C14—H14*B*⋯O3	0.96	2.40	3.280 (6)	152
O5—H5⋯O1^i^	0.82	2.13	2.908 (7)	158

Symmetry code: (i) 
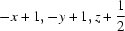
.

**Fig. 1.**
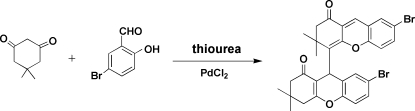
palladium(II) chloride catalyzed synthesis of the title compound.

**Fig. 2.**
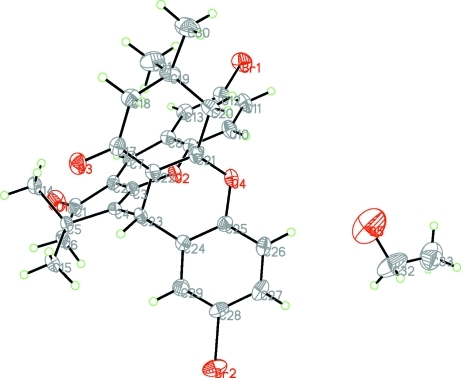
View of the title compound showing the atom-labelling scheme. Ellipsoids are drawn at the 50% probability level.

**Fig. 3.**
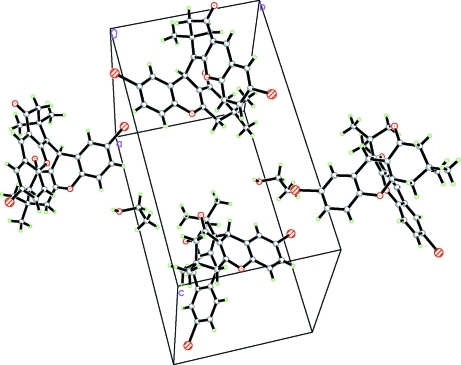
The perspective packing view of the titie compound.

## supplementary crystallographic information

### Comment

Organic molecules containing xanthene are of biological importance and useful in drug discovery. They are also very efficient laser dyes and outstanding photophysical properties. (Gusak *et al.*, 2000; Sato *et al.*, 2008; Wang *et al.*, 2005). As one part of our on going studies on the synthesis of xanthene-containing heterocyclic compounds, the title compound was isolated unexpectedly.

The reaction between 5-bromosalicybenzaldehyde and 5,5-dimethyl-1,3-cyclohexanedione proceeded to give the title compound in excellent yield in the presence of palladium(II) chloride as catalyst in acetonitrile under reflux (Figure 1). The structure of the title compound is illustrated in Figure 2. There are no unusual bond lengths and angles in the compound. The phenyl rings show no interactions in the crystal. The compound contains two similar linked rings: a pyranoid ring connected two six-membered rings, the structure about two big rings connected each other *via* C3—C23 bond. The two big rings are approximately planar (Figure 3). In addition, the molecule is connected with the ethanol molecule by O(5)—H(5)···O(1) and interior C(14)—H(14)···O(3), which is less than the sum of their van der Waals.

### Experimental

A mixture of 5-bromosalicybenzaldehyde (1.00g, 5mmol), 5,5-dimethyl-1,3-cyclohexanedione (1.12 g, 10 mmol), thiourea (0.76 g, 10 mmol), and palladium(II) chloride was refluxed in acetonitrile (12 ml) at 298 K for 12 h. After being cooled to room temperature, The red precipitation was filtered through a silica pad, and then washed twice with water, dried under vacuum to yield the product. Single crystal of the title compound was obtained by slow evaporation from ethanol at room temperature. The compound is isolated as red, block-shaped crystal from ethanol at room temperature.

### Refinement

The H atoms were positioned geometrically and allowed to ride on their parentatoms, with C—H = 0.93–0.97 Å, and O—H = 0.82 Å and *U*_iso_ = 1.2 or 1.5 U_eq_(parent atom).

### Crystal data

**Table:**

C_30_H_26_Br_2_O_4_·C_2_H_6_O	*F*(000) = 1336
*M**_r_* = 656.40	*D*_x_ = 1.512 Mg m^−^^3^
Orthorhombic, *P**c**a*2_1_	Mo *K*α radiation, λ = 0.71073 Å
Hall symbol: P 2c -2ac	Cell parameters from 3559 reflections
*a* = 15.016 (2) Å	θ = 2.2–21.8°
*b* = 10.2459 (14) Å	µ = 2.85 mm^−^^1^
*c* = 18.740 (3) Å	*T* = 298 K
*V* = 2883.2 (7) Å^3^	Block, red
*Z* = 4	0.26 × 0.18 × 0.12 mm

### Data collection

**Table:**

Bruker APEXII area-detector diffractometer	5155 independent reflections
Radiation source: fine-focus sealed tube	3685 reflections with *I* > 2σ(*I*)
graphite	*R*_int_ = 0.041
φ and ω scan	θ_max_ = 25.5°, θ_min_ = 2.2°
Absorption correction: multi-scan (*SADABS*; Bruker, 2004)	*h* = −18→17
*T*_min_ = 0.546, *T*_max_ = 0.710	*k* = −11→12
14442 measured reflections	*l* = −22→22

### Refinement

**Table:**

Refinement on *F*^2^	Secondary atom site location: difference Fourier map
Least-squares matrix: full	Hydrogen site location: inferred from neighbouring sites
*R*[*F*^2^ > 2σ(*F*^2^)] = 0.039	H-atom parameters constrained
*wR*(*F*^2^) = 0.078	*w* = 1/[σ^2^(*F*_o_^2^) + (0.016*P*)^2^ + 0.6332*P*] where *P* = (*F*_o_^2^ + 2*F*_c_^2^)/3
*S* = 1.00	(Δ/σ)_max_ = 0.001
5155 reflections	Δρ_max_ = 0.33 e Å^−^^3^
357 parameters	Δρ_min_ = −0.27 e Å^−^^3^
1 restraint	Absolute structure: Flack (1983), 2379 Friedel pairs
Primary atom site location: structure-invariant direct methods	Flack parameter: 0.020 (8)

### Special details

**Table:**

Geometry. All esds (except the esd in the dihedral angle between two l.s. planes) are estimated using the full covariance matrix. The cell esds are taken into account individually in the estimation of esds in distances, angles and torsion angles; correlations between esds in cell parameters are only used when they are defined by crystal symmetry. An approximate (isotropic) treatment of cell esds is used for estimating esds involving l.s. planes.
Refinement. Refinement of F^2^ against ALL reflections. The weighted R-factor wR and goodness of fit S are based on F^2^, conventional R-factors R are based on F, with F set to zero for negative F^2^. The threshold expression of F^2^ > 2sigma(F^2^) is used only for calculating R-factors(gt) etc. and is not relevant to the choice of reflections for refinement. R-factors based on F^2^ are statistically about twice as large as those based on F, and R- factors based on ALL data will be even larger.

### Fractional atomic coordinates and isotropic or equivalent isotropic displacement parameters (Å^2^)

**Table:**

	*x*	*y*	*z*	*U*_iso_*/*U*_eq_	
Br1	0.42195 (3)	0.14209 (5)	0.41954 (3)	0.06989 (17)	
Br2	0.62250 (4)	1.02446 (4)	0.12373 (4)	0.07602 (18)	
C9	0.5865 (3)	0.3725 (4)	0.2527 (2)	0.0339 (10)	
C2	0.5465 (3)	0.3620 (4)	0.1083 (2)	0.0343 (9)	
C8	0.5154 (3)	0.2968 (4)	0.2299 (2)	0.0352 (9)	
C3	0.6267 (2)	0.4279 (3)	0.1314 (2)	0.0332 (9)	
C4	0.6893 (3)	0.4756 (4)	0.08862 (18)	0.0297 (9)	
C6	0.5783 (3)	0.4647 (5)	−0.0097 (2)	0.0475 (11)	
H6A	0.5590	0.5534	−0.0003	0.057*	
H6B	0.5701	0.4480	−0.0602	0.057*	
C7	0.4942 (3)	0.2991 (4)	0.1543 (2)	0.0368 (10)	
H7	0.4436	0.2562	0.1378	0.044*	
C5	0.6777 (3)	0.4528 (4)	0.00836 (18)	0.0380 (10)	
C12	0.4897 (3)	0.2371 (4)	0.3510 (2)	0.0468 (11)	
C13	0.4668 (3)	0.2280 (4)	0.2804 (2)	0.0427 (10)	
H13	0.4190	0.1761	0.2664	0.051*	
C11	0.5580 (3)	0.3144 (5)	0.3737 (2)	0.0483 (12)	
H11	0.5717	0.3205	0.4219	0.058*	
C1	0.5220 (3)	0.3733 (4)	0.0316 (2)	0.0452 (11)	
C10	0.6068 (3)	0.3839 (4)	0.3235 (2)	0.0451 (11)	
H10	0.6531	0.4381	0.3380	0.054*	
O1	0.4611 (3)	0.3130 (4)	0.00624 (17)	0.0721 (10)	
C14	0.7095 (3)	0.3150 (4)	−0.0113 (2)	0.0480 (11)	
H14A	0.6741	0.2516	0.0136	0.072*	
H14B	0.7708	0.3051	0.0021	0.072*	
H14C	0.7034	0.3021	−0.0618	0.072*	
C15	0.7300 (3)	0.5510 (4)	−0.0373 (2)	0.0512 (12)	
H15A	0.7118	0.5435	−0.0862	0.077*	
H15B	0.7926	0.5329	−0.0335	0.077*	
H15C	0.7184	0.6379	−0.0206	0.077*	
O2	0.63770 (16)	0.4421 (3)	0.20540 (13)	0.0380 (7)	
C21	0.8334 (3)	0.4733 (4)	0.2348 (2)	0.0333 (9)	
C24	0.7435 (3)	0.6669 (4)	0.1613 (2)	0.0339 (9)	
C17	0.8935 (3)	0.3742 (4)	0.1292 (2)	0.0381 (9)	
C23	0.7709 (2)	0.5480 (3)	0.1187 (2)	0.0324 (8)	
H23	0.8062	0.5787	0.0780	0.039*	
C28	0.6787 (3)	0.8803 (4)	0.1690 (3)	0.0462 (11)	
C29	0.7040 (2)	0.7750 (4)	0.1286 (2)	0.0440 (10)	
H29	0.6949	0.7755	0.0796	0.053*	
C22	0.8303 (3)	0.4641 (4)	0.1636 (2)	0.0320 (9)	
C25	0.7517 (3)	0.6708 (4)	0.2340 (2)	0.0360 (9)	
C27	0.6889 (3)	0.8824 (4)	0.2422 (3)	0.0515 (12)	
H27	0.6710	0.9546	0.2687	0.062*	
C26	0.7258 (3)	0.7757 (4)	0.2750 (2)	0.0417 (10)	
H26	0.7333	0.7743	0.3243	0.050*	
C20	0.8834 (3)	0.3859 (4)	0.2829 (2)	0.0431 (11)	
H20A	0.8475	0.3686	0.3249	0.052*	
H20B	0.9372	0.4301	0.2984	0.052*	
C19	0.9089 (3)	0.2562 (4)	0.2483 (2)	0.0452 (11)	
C18	0.9498 (3)	0.2880 (4)	0.1766 (2)	0.0465 (11)	
H18A	1.0066	0.3307	0.1844	0.056*	
H18B	0.9615	0.2068	0.1516	0.056*	
O3	0.9023 (2)	0.3738 (3)	0.06442 (16)	0.0526 (8)	
O4	0.78986 (17)	0.5671 (3)	0.27188 (12)	0.0382 (7)	
C30	0.9769 (4)	0.1859 (5)	0.2962 (3)	0.0721 (15)	
H30A	0.9936	0.1046	0.2746	0.108*	
H30B	0.9508	0.1696	0.3421	0.108*	
H30C	1.0288	0.2397	0.3018	0.108*	
C31	0.8274 (4)	0.1713 (5)	0.2391 (3)	0.0683 (14)	
H31A	0.7836	0.2173	0.2115	0.102*	
H31B	0.8032	0.1503	0.2851	0.102*	
H31C	0.8437	0.0923	0.2148	0.102*	
O5	0.6800 (4)	0.8520 (6)	0.4501 (3)	0.169 (3)	
H5	0.6333	0.8118	0.4557	0.253*	
C33	0.6290 (5)	1.0123 (7)	0.5278 (4)	0.113 (2)	
H33A	0.6304	0.9339	0.5558	0.170*	
H33B	0.5688	1.0428	0.5239	0.170*	
H33C	0.6648	1.0780	0.5504	0.170*	
C32	0.6640 (6)	0.9856 (7)	0.4571 (5)	0.121 (3)	
H32A	0.6215	1.0137	0.4212	0.145*	
H32B	0.7189	1.0337	0.4499	0.145*	

### Atomic displacement parameters (Å^2^)

**Table:**

	*U*^11^	*U*^22^	*U*^33^	*U*^12^	*U*^13^	*U*^23^
Br1	0.0664 (3)	0.0769 (4)	0.0663 (3)	−0.0076 (3)	0.0261 (3)	0.0185 (3)
Br2	0.0842 (4)	0.0433 (3)	0.1006 (4)	0.0189 (3)	−0.0024 (3)	0.0077 (3)
C9	0.028 (2)	0.037 (2)	0.037 (2)	0.0032 (19)	0.0031 (18)	0.001 (2)
C2	0.034 (2)	0.036 (2)	0.033 (2)	0.0049 (18)	−0.0022 (18)	−0.0084 (19)
C8	0.025 (2)	0.040 (2)	0.040 (2)	0.0040 (18)	0.0026 (18)	0.0015 (19)
C3	0.037 (2)	0.036 (2)	0.027 (2)	0.0033 (17)	−0.0050 (18)	−0.002 (2)
C4	0.034 (2)	0.027 (2)	0.0283 (19)	0.0044 (18)	−0.0014 (17)	0.0016 (16)
C6	0.053 (3)	0.060 (3)	0.029 (2)	0.014 (2)	−0.010 (2)	−0.002 (2)
C7	0.032 (2)	0.035 (2)	0.044 (2)	0.0044 (18)	−0.0063 (19)	−0.0096 (18)
C5	0.049 (3)	0.040 (3)	0.025 (2)	0.007 (2)	−0.0034 (18)	−0.0027 (17)
C12	0.040 (3)	0.055 (3)	0.046 (3)	0.000 (2)	0.015 (2)	0.009 (2)
C13	0.031 (2)	0.046 (3)	0.052 (3)	0.002 (2)	0.003 (2)	−0.001 (2)
C11	0.044 (3)	0.071 (3)	0.029 (2)	0.002 (2)	0.003 (2)	0.002 (2)
C1	0.041 (3)	0.052 (3)	0.043 (2)	0.005 (2)	−0.010 (2)	−0.011 (2)
C10	0.038 (3)	0.060 (3)	0.038 (2)	−0.006 (2)	0.0007 (19)	−0.001 (2)
O1	0.070 (2)	0.088 (3)	0.058 (2)	−0.022 (2)	−0.0222 (18)	−0.0086 (19)
C14	0.061 (3)	0.050 (3)	0.033 (2)	0.011 (2)	−0.003 (2)	−0.006 (2)
C15	0.069 (4)	0.058 (3)	0.026 (2)	0.001 (2)	0.001 (2)	0.002 (2)
O2	0.0399 (17)	0.0495 (17)	0.0245 (13)	−0.0103 (13)	0.0010 (13)	−0.0024 (13)
C21	0.028 (2)	0.040 (2)	0.031 (2)	−0.0013 (19)	0.0027 (18)	−0.0038 (19)
C24	0.030 (2)	0.036 (2)	0.036 (2)	−0.0031 (19)	0.0044 (17)	0.000 (2)
C17	0.034 (2)	0.042 (2)	0.039 (2)	−0.0026 (17)	0.0072 (19)	−0.004 (2)
C23	0.032 (2)	0.040 (2)	0.0245 (18)	−0.0007 (16)	0.0041 (18)	0.001 (2)
C28	0.042 (3)	0.032 (2)	0.065 (3)	0.005 (2)	0.004 (2)	0.001 (2)
C29	0.047 (3)	0.042 (2)	0.043 (2)	−0.0034 (19)	0.004 (2)	0.000 (2)
C22	0.027 (2)	0.035 (2)	0.034 (2)	0.0000 (18)	0.0043 (17)	0.0004 (18)
C25	0.034 (2)	0.034 (2)	0.040 (2)	−0.0039 (18)	−0.0020 (19)	−0.003 (2)
C27	0.046 (3)	0.043 (3)	0.065 (3)	−0.004 (2)	0.009 (2)	−0.020 (2)
C26	0.040 (3)	0.045 (3)	0.040 (2)	−0.005 (2)	0.0067 (19)	−0.012 (2)
C20	0.037 (2)	0.054 (3)	0.039 (2)	−0.003 (2)	−0.0022 (19)	0.011 (2)
C19	0.034 (3)	0.045 (3)	0.056 (3)	0.001 (2)	−0.005 (2)	0.011 (2)
C18	0.037 (3)	0.050 (3)	0.052 (3)	0.007 (2)	0.000 (2)	0.004 (2)
O3	0.051 (2)	0.064 (2)	0.0423 (19)	0.0083 (15)	0.0145 (14)	−0.0049 (15)
O4	0.0409 (18)	0.0456 (17)	0.0281 (14)	0.0005 (14)	0.0014 (12)	−0.0047 (13)
C30	0.065 (4)	0.082 (4)	0.070 (3)	0.022 (3)	−0.004 (3)	0.021 (3)
C31	0.065 (4)	0.050 (3)	0.091 (4)	−0.014 (3)	0.004 (3)	0.010 (3)
O5	0.164 (5)	0.141 (5)	0.200 (7)	−0.022 (4)	0.093 (5)	−0.032 (4)
C33	0.128 (7)	0.107 (6)	0.105 (5)	0.011 (5)	0.003 (5)	−0.029 (4)
C32	0.129 (6)	0.065 (5)	0.169 (8)	0.023 (4)	0.035 (5)	−0.014 (5)

### Geometric parameters (Å, °)

**Table:**

Br1—C12	1.906 (4)	C24—C29	1.398 (5)
Br2—C28	1.901 (4)	C24—C23	1.514 (5)
C9—C10	1.366 (6)	C17—O3	1.221 (5)
C9—O2	1.374 (5)	C17—C22	1.471 (5)
C9—C8	1.387 (5)	C17—C18	1.512 (6)
C2—C7	1.332 (5)	C23—C22	1.498 (5)
C2—C3	1.447 (5)	C23—H23	0.9800
C2—C1	1.488 (6)	C28—C29	1.371 (5)
C8—C13	1.388 (5)	C28—C27	1.381 (6)
C8—C7	1.453 (5)	C29—H29	0.9300
C3—C4	1.328 (5)	C25—C26	1.377 (5)
C3—O2	1.404 (4)	C25—O4	1.401 (5)
C4—C5	1.532 (5)	C27—C26	1.371 (6)
C4—C23	1.540 (5)	C27—H27	0.9300
C6—C1	1.481 (6)	C26—H26	0.9300
C6—C5	1.535 (6)	C20—C19	1.527 (6)
C6—H6A	0.9700	C20—H20A	0.9700
C6—H6B	0.9700	C20—H20B	0.9700
C7—H7	0.9300	C19—C31	1.512 (6)
C5—C14	1.535 (6)	C19—C18	1.513 (6)
C5—C15	1.537 (6)	C19—C30	1.539 (6)
C12—C11	1.364 (6)	C18—H18A	0.9700
C12—C13	1.371 (6)	C18—H18B	0.9700
C13—H13	0.9300	C30—H30A	0.9600
C11—C10	1.388 (6)	C30—H30B	0.9600
C11—H11	0.9300	C30—H30C	0.9600
C1—O1	1.202 (5)	C31—H31A	0.9600
C10—H10	0.9300	C31—H31B	0.9600
C14—H14A	0.9600	C31—H31C	0.9600
C14—H14B	0.9600	O5—C32	1.397 (8)
C14—H14C	0.9600	O5—H5	0.8200
C15—H15A	0.9600	C33—C32	1.451 (10)
C15—H15B	0.9600	C33—H33A	0.9600
C15—H15C	0.9600	C33—H33B	0.9600
C21—C22	1.338 (5)	C33—H33C	0.9600
C21—O4	1.354 (5)	C32—H32A	0.9700
C21—C20	1.476 (5)	C32—H32B	0.9700
C24—C25	1.368 (6)		
			
C10—C9—O2	117.2 (4)	C22—C23—C4	113.8 (3)
C10—C9—C8	121.3 (4)	C24—C23—C4	111.4 (3)
O2—C9—C8	121.4 (3)	C22—C23—H23	107.4
C7—C2—C3	121.6 (3)	C24—C23—H23	107.4
C7—C2—C1	121.1 (4)	C4—C23—H23	107.4
C3—C2—C1	117.2 (4)	C29—C28—C27	122.0 (4)
C9—C8—C13	118.6 (4)	C29—C28—Br2	119.3 (3)
C9—C8—C7	117.5 (4)	C27—C28—Br2	118.7 (3)
C13—C8—C7	123.9 (4)	C28—C29—C24	120.0 (4)
C4—C3—O2	118.3 (3)	C28—C29—H29	120.0
C4—C3—C2	125.5 (3)	C24—C29—H29	120.0
O2—C3—C2	116.2 (3)	C21—C22—C17	117.4 (4)
C3—C4—C5	117.1 (3)	C21—C22—C23	122.7 (4)
C3—C4—C23	121.3 (3)	C17—C22—C23	119.8 (3)
C5—C4—C23	121.6 (3)	C24—C25—C26	123.6 (4)
C1—C6—C5	112.9 (3)	C24—C25—O4	121.3 (4)
C1—C6—H6A	109.0	C26—C25—O4	115.1 (3)
C5—C6—H6A	109.0	C26—C27—C28	118.6 (4)
C1—C6—H6B	109.0	C26—C27—H27	120.7
C5—C6—H6B	109.0	C28—C27—H27	120.7
H6A—C6—H6B	107.8	C27—C26—C25	119.0 (4)
C2—C7—C8	120.6 (4)	C27—C26—H26	120.5
C2—C7—H7	119.7	C25—C26—H26	120.5
C8—C7—H7	119.7	C21—C20—C19	113.4 (3)
C4—C5—C6	108.4 (3)	C21—C20—H20A	108.9
C4—C5—C14	109.9 (3)	C19—C20—H20A	108.9
C6—C5—C14	108.8 (3)	C21—C20—H20B	108.9
C4—C5—C15	112.9 (3)	C19—C20—H20B	108.9
C6—C5—C15	108.8 (3)	H20A—C20—H20B	107.7
C14—C5—C15	108.0 (3)	C31—C19—C18	110.5 (4)
C11—C12—C13	121.9 (4)	C31—C19—C20	110.3 (4)
C11—C12—Br1	119.3 (3)	C18—C19—C20	107.0 (3)
C13—C12—Br1	118.8 (3)	C31—C19—C30	109.5 (4)
C12—C13—C8	119.5 (4)	C18—C19—C30	110.5 (4)
C12—C13—H13	120.3	C20—C19—C30	109.1 (4)
C8—C13—H13	120.3	C17—C18—C19	114.9 (3)
C12—C11—C10	119.0 (4)	C17—C18—H18A	108.6
C12—C11—H11	120.5	C19—C18—H18A	108.6
C10—C11—H11	120.5	C17—C18—H18B	108.6
O1—C1—C6	123.5 (4)	C19—C18—H18B	108.6
O1—C1—C2	122.0 (4)	H18A—C18—H18B	107.5
C6—C1—C2	114.5 (4)	C21—O4—C25	118.4 (3)
C9—C10—C11	119.7 (4)	C19—C30—H30A	109.5
C9—C10—H10	120.2	C19—C30—H30B	109.5
C11—C10—H10	120.2	H30A—C30—H30B	109.5
C5—C14—H14A	109.5	C19—C30—H30C	109.5
C5—C14—H14B	109.5	H30A—C30—H30C	109.5
H14A—C14—H14B	109.5	H30B—C30—H30C	109.5
C5—C14—H14C	109.5	C19—C31—H31A	109.5
H14A—C14—H14C	109.5	C19—C31—H31B	109.5
H14B—C14—H14C	109.5	H31A—C31—H31B	109.5
C5—C15—H15A	109.5	C19—C31—H31C	109.5
C5—C15—H15B	109.5	H31A—C31—H31C	109.5
H15A—C15—H15B	109.5	H31B—C31—H31C	109.5
C5—C15—H15C	109.5	C32—O5—H5	109.5
H15A—C15—H15C	109.5	C32—C33—H33A	109.5
H15B—C15—H15C	109.5	C32—C33—H33B	109.5
C9—O2—C3	121.2 (3)	H33A—C33—H33B	109.5
C22—C21—O4	123.1 (4)	C32—C33—H33C	109.5
C22—C21—C20	125.6 (4)	H33A—C33—H33C	109.5
O4—C21—C20	111.3 (3)	H33B—C33—H33C	109.5
C25—C24—C29	116.8 (4)	O5—C32—C33	109.4 (6)
C25—C24—C23	121.6 (3)	O5—C32—H32A	109.8
C29—C24—C23	121.5 (4)	C33—C32—H32A	109.8
O3—C17—C22	120.6 (4)	O5—C32—H32B	109.8
O3—C17—C18	121.4 (4)	C33—C32—H32B	109.8
C22—C17—C18	118.0 (4)	H32A—C32—H32B	108.2
C22—C23—C24	109.1 (3)		
			
C10—C9—C8—C13	−2.4 (6)	C25—C24—C23—C4	109.0 (4)
O2—C9—C8—C13	179.5 (3)	C29—C24—C23—C4	−67.3 (5)
C10—C9—C8—C7	174.9 (4)	C3—C4—C23—C22	64.4 (5)
O2—C9—C8—C7	−3.1 (6)	C5—C4—C23—C22	−114.3 (4)
C7—C2—C3—C4	169.2 (4)	C3—C4—C23—C24	−59.5 (5)
C1—C2—C3—C4	−14.0 (6)	C5—C4—C23—C24	121.9 (4)
C7—C2—C3—O2	−11.2 (5)	C27—C28—C29—C24	−1.7 (6)
C1—C2—C3—O2	165.6 (3)	Br2—C28—C29—C24	−178.5 (3)
O2—C3—C4—C5	177.2 (3)	C25—C24—C29—C28	2.5 (6)
C2—C3—C4—C5	−3.2 (6)	C23—C24—C29—C28	179.0 (4)
O2—C3—C4—C23	−1.6 (5)	O4—C21—C22—C17	168.7 (3)
C2—C3—C4—C23	178.1 (3)	C20—C21—C22—C17	−11.0 (6)
C3—C2—C7—C8	1.2 (6)	O4—C21—C22—C23	−6.6 (6)
C1—C2—C7—C8	−175.5 (4)	C20—C21—C22—C23	173.7 (4)
C9—C8—C7—C2	6.2 (6)	O3—C17—C22—C21	−170.3 (4)
C13—C8—C7—C2	−176.6 (4)	C18—C17—C22—C21	6.7 (5)
C3—C4—C5—C6	37.6 (5)	O3—C17—C22—C23	5.1 (6)
C23—C4—C5—C6	−143.6 (3)	C18—C17—C22—C23	−177.9 (3)
C3—C4—C5—C14	−81.2 (4)	C24—C23—C22—C21	19.4 (5)
C23—C4—C5—C14	97.5 (4)	C4—C23—C22—C21	−105.7 (4)
C3—C4—C5—C15	158.2 (4)	C24—C23—C22—C17	−155.8 (3)
C23—C4—C5—C15	−23.0 (5)	C4—C23—C22—C17	79.1 (4)
C1—C6—C5—C4	−57.1 (4)	C29—C24—C25—C26	−2.1 (6)
C1—C6—C5—C14	62.4 (4)	C23—C24—C25—C26	−178.5 (4)
C1—C6—C5—C15	179.9 (3)	C29—C24—C25—O4	179.7 (3)
C11—C12—C13—C8	1.5 (7)	C23—C24—C25—O4	3.2 (6)
Br1—C12—C13—C8	179.9 (3)	C29—C28—C27—C26	0.3 (7)
C9—C8—C13—C12	0.3 (6)	Br2—C28—C27—C26	177.1 (3)
C7—C8—C13—C12	−176.9 (4)	C28—C27—C26—C25	0.2 (6)
C13—C12—C11—C10	−1.2 (7)	C24—C25—C26—C27	0.8 (6)
Br1—C12—C11—C10	−179.7 (3)	O4—C25—C26—C27	179.1 (4)
C5—C6—C1—O1	−138.1 (4)	C22—C21—C20—C19	−18.5 (6)
C5—C6—C1—C2	42.3 (5)	O4—C21—C20—C19	161.8 (3)
C7—C2—C1—O1	−9.7 (6)	C21—C20—C19—C31	−71.7 (5)
C3—C2—C1—O1	173.5 (4)	C21—C20—C19—C18	48.5 (4)
C7—C2—C1—C6	169.9 (4)	C21—C20—C19—C30	168.0 (4)
C3—C2—C1—C6	−6.9 (5)	O3—C17—C18—C19	−156.0 (4)
O2—C9—C10—C11	−179.1 (4)	C22—C17—C18—C19	27.0 (5)
C8—C9—C10—C11	2.7 (6)	C31—C19—C18—C17	67.1 (5)
C12—C11—C10—C9	−0.9 (7)	C20—C19—C18—C17	−53.0 (5)
C10—C9—O2—C3	174.5 (3)	C30—C19—C18—C17	−171.6 (4)
C8—C9—O2—C3	−7.3 (5)	C22—C21—O4—C25	−10.4 (6)
C4—C3—O2—C9	−166.1 (3)	C20—C21—O4—C25	169.3 (3)
C2—C3—O2—C9	14.2 (5)	C24—C25—O4—C21	11.9 (5)
C25—C24—C23—C22	−17.4 (5)	C26—C25—O4—C21	−166.5 (3)
C29—C24—C23—C22	166.3 (4)		

### Hydrogen-bond geometry (Å, °)

**Table:**

*D*—H···*A*	*D*—H	H···*A*	*D*···*A*	*D*—H···*A*
C14—H14B···O3	0.96	2.40	3.280 (6)	152
O5—H5···O1^i^	0.82	2.13	2.908 (7)	158

Symmetry codes: (i) −*x*+1, −*y*+1, *z*+1/2.
